# The retinoic acid receptor-α modulators ATRA and Ro415253 reciprocally regulate human IL-5+ Th2 cell proliferation and cytokine expression

**DOI:** 10.1186/1476-7961-11-4

**Published:** 2013-12-06

**Authors:** Daniel L Wansley, Yuzhi Yin, Calman Prussin

**Affiliations:** 1 National Institute of Allergy and Infectious Disease, National Institutes of Health, Laboratory of Allergic Diseases, NIH, 10 Center Drive, MSC-1881, Bethesda, MD, 20852-1881, USA

**Keywords:** Interleukin 5, All-trans retinoic acid, T helper 2, Retinoic acid receptor alpha, House dust mite antigen, Retinoic acid response element

## Abstract

**Background:**

Th2 cytokine responses are enhanced by all trans retinoic acid (ATRA), the bioavailable form of vitamin A. Retinoic acid receptor alpha (RARα) is the high affinity receptor for ATRA that mediates these pro-Th2 effects. We have previously characterized two major human Th2 subpopulations: IL-5- Th2 (IL-5-, IL-4+, IL-13+) and IL-5+ Th2 cells (IL-5+, IL-4+, IL-13+), which represent less and more highly differentiated Th2 cells, respectively. We hypothesized that the pro-Th2 effects of ATRA may differentially affect these Th2 subpopulations.

**Methods:**

Specific cytokine producing Th2 subpopulations were identified using intracellular cytokine staining. Proliferation was measured using the Cell Trace Violet proliferation tracking dye. Apoptotic cells were identified using either annexin-V or active caspase 3 staining. Th2 gene expression was measured using quantitative polymerase chain reaction.

**Results:**

ATRA increased the output of Th2 cells from house dust mite allergen (HDM) specific short-term cell lines, and this enhancement was limited to the IL-5+ Th2 subpopulation. Conversely, the RARα antagonist Ro415253 decreased Th2 cell output from these cultures, and this effect was again limited to the IL-5+ Th2 subpopulation. ATRA and Ro415253 respectively augmented and inhibited Th2 cell proliferation, and this affect was more pronounced for the IL-5+ vs. IL-5- Th2 subpopulation. ATRA and Ro415253 respectively augmented and inhibited the expression of IL5 in a significant manner, which was not found for IL4 or IL13.

**Conclusions:**

We report that the reciprocal regulation of Th2 cytokine expression and proliferation by RARα modulators are largely limited to modulation of IL-5 gene expression and to proliferation of the highly differentiated IL-5+ Th2 subpopulation. These results suggest that RARα antagonism is a potential means to therapeutically target allergic inflammation.

**Trial registration:**

Clinicaltrials.gov identifier: NCT01212016

## Background

The association between vitamin A and effective immune responses dates back to the early 20th century when vitamin A was described as an “anti-infective agent” [1,2]. Large placebo controlled studies in vitamin deficient populations in developing countries show that vitamin A supplementation is associated with decreased childhood mortality from diarrheal disease and measles, underscoring the critical role for vitamin A in immunity. Studies on vitamin A deficiency have consistently demonstrated an indispensable role for vitamin A in maintaining host immunity to a variety of pathogens [3-5].

Retinoic acid (RA) is the bioactive form of vitamin A; all-trans-retinoic acid (ATRA) is the most abundant form of RA found in the circulation. RA influences immunity via multiple mechanisms. CD4 T cells are the immune component most prominently affected by RA. RA augments the differentiation of inducible T regulatory cells, which is abrogated both in vitro and in vivo in either vitamin A deficiency states or using retinoic acid receptor (RAR) deficient mice [5]. In vitamin A deficient mice, RA restores CD4+ T cell-mediated immunity, homeostasis, and activation [4,5]. Previous investigations suggest that retinoic acid receptor alpha (RARα) mediates these RA-induced effects on T cells [5-8]. ATRA exerts its bioactivity via binding to retinoic acid receptor ligand-activated transcription factors, and ATRA is a high-affinity ligand for retinoic acid receptor alpha (RARα). ATRA binding to RAR monomers then induces hetero-dimerization with retinoid X receptors (RXRs). The RAR-RXR complex in turn binds to retinoic acid response elements (RARE) which are present in the promoters of RA responsive genes and result in gene activation [9].

Th2 cells were initially characterized as expressing IL-4, IL-5, and IL-13 [10]. IL-4 is the major factor driving Th2 differentiation, IgE class switching, and alternative macrophage activation, whereas IL-13 functions as an effector molecule that mediates eosinophilic inflammation, airway hyper-responsiveness, and mucus secretion. IL-5 is the major eosinophil-active cytokine and induces eosinophilopoiesis and eosinophil release from the bone marrow, enhances eosinophil survival, and acts as a costimulator for eosinophil activation [11,12]. In vivo, vitamin A is associated with eosinophilic tissue inflammation, which is both a protective component of anti-helminth immunity and a major contributor to asthma pathogenesis. Vitamin A deficiency inhibits parasite expulsion due to reduced eosinophilia and IL-5 secretion by antigen-specific lymphocytes in vivo, and vitamin A supplementation restores parasite immunity [11-14]. Vitamin A deficiency diminishes and high-level vitamin A supplementation restores Th2 cytokines and eosinophilia induced by experimental asthma [15].

RARα agonists and RARα antagonists exert opposite effects on the production of Th2 cytokines by in vitro stimulated T cells [6,16]. We have recently characterized two major subpopulations within the Th2 lineage: IL-5+ Th2, which express IL-5, IL-4, and IL-13, and IL-5- Th2 cells, which only express the latter two cytokines [17]. IL-5+ Th2 cells are more highly differentiated and have greater pro-inflammatory activity than IL-5- Th2 cells [18,19]. IL-5+ Th2 cells are tightly linked to blood eosinophilia [20]. Given the known pro-Th2 activity of RA, we sought to determine if RA drives the differentiation of IL-5- Th2 to IL-5+ Th2 cells or otherwise enhances the generation of IL-5+ Th2 cells. Using a variety of human in vitro Th2 model systems, in this work we demonstrate the reciprocal regulation of cytokine production and proliferation by RARα modulators are specific for the IL-5 gene and these effects are limited to the highly differentiated IL-5+ Th2 cell subpopulation.

## Materials and methods

### Subjects

Subjects underwent lymphapheresis (National Institutes of Health Clinical Center Department of Transfusion Medicine), and PBMC were isolated as described [17]. Donors used for lymphapheresis included healthy non-allergic control, eosinophilic gastrointestinal disease and allergic asthmatic subjects. Allergic asthmatic subjects had a minimum one-year history of episodic bronchospasm relieved by β-agonist medications and three or more positive skin test responses (≥3 mm) out of a panel of 10 aeroallergens. The National Institute of Allergy and Infectious Diseases Institutional Review Board approved the clinical protocols used for this study. All subjects signed informed consent.

### Cells and culture

For Th2 differentiation, naïve T cells were obtained from PBMC using the naïve CD4+ T cell isolation kit (Miltenyi Biotec, Auburn, CA). Naïve cells were Th2 polarized as published [17]. Th2 polarization cycles were repeated at weekly intervals, where 1×Th2, 2×Th2, and 3×Th2 represent 1, 2, and 3 serial 7d cycles of differentiation. For each 7 d round of stimulation, ATRA/vehicle was added on day 1 and, as replenishment, on day 4 of culture. After differentiation, Th2-polarized cells were cryopreserved in liquid N_2_, and subsequently thawed and recovered in complete media containing IL-2 (50–100 U/ml) for at least 24 h prior to RA experiments.

**Figure 1:**
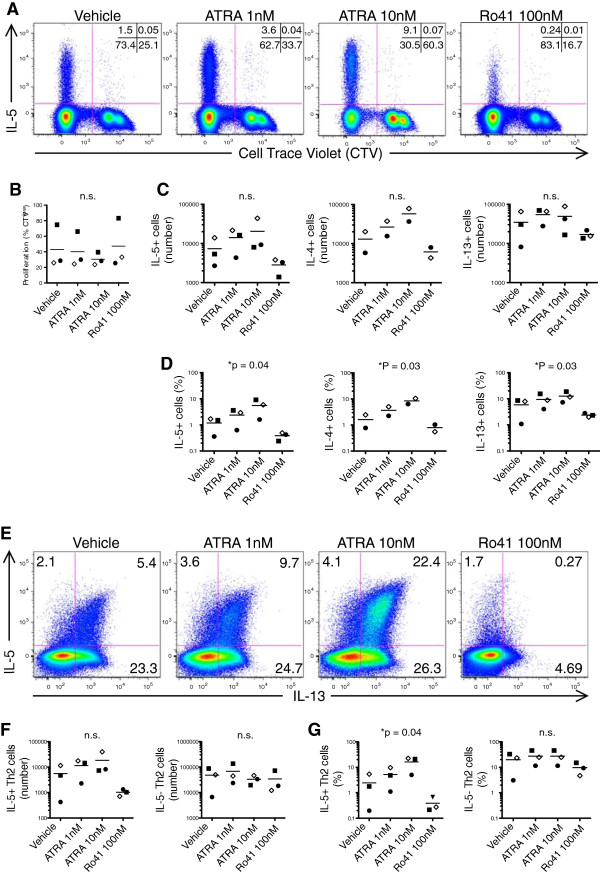
**Enhancement of Th2 cell output in HDM-stimulated PBMC cultures is reciprocally regulated by RARα modulators.** Cell trace violet (CTV)-labeled PBMCs from allergic asthmatic subjects were stimulated with HDM Ag extract (40 U/ml) for 10d in the presence of ATRA (RARα agonist), Ro41 (RARα antagonist), or DMSO vehicle control. After 10d cultures were restimulated with PMA and ionomycin and ICCS was performed. **(A)** Representative flow plots are shown. Combined results from 3 cultures showing **(B)** the frequency of CTV^low^ cells, after gating on viable HDM proliferated CD3+, CD4+, CD8- cells. Combined results from 3 cultures showing **(C)** the cell number and **(D)** percentage of Th2 cytokine producing HDM-expanded T cells. **(E)** Representative flow plots showing IL-13 vs. IL-5 expression after gating on viable HDM proliferated CD3^+^ CD4^+^ CTV^low^ cells. Combined results from 3 cultures showing **(F)** cell number and **(G)** percentage of of IL-5^+^ (IL-5^+^ IL-13^+^) and IL-5^-^ (IL-5^-^ IL-13^+^) Th2 cell populations. Data points represent independent HDM-stimulated PBMC cultures. P-values were generated with 2-way ANOVA. In B and C, only 2 experiments were performed using IL-4 as an analyte.

For proliferation assays, PBMC and Th2-polarized cells were labeled with 5 μM of Cell Trace Violet proliferation tracking dye (CTV; Invitrogen, Carlsbad, CA) according to instructions. Using PBMC, 10d cultures were used to observe optimal proliferation; in such prolonged proliferation experiments, both the media control (data not shown) as well as the allergen activated cultures yielded 2 CTV bright “negative” peaks (Figure 1A, lower right hand quadrant). Using 12d antigen activated cultures, similar doublet “negative” peaks were described by Givan and Wallace [21,22] and are thought to be an artifact due to homeostatic proliferation during the prolonged culture.

PBMC proliferation assays were performed using complete media containing 5% heat inactivated human AB serum (Invitrogen). Th2 cell proliferation assays were performed with complete media containing 10% heat inactivated FBS (Invitrogen). Th2 cell cultures used for qRT-PCR were performed using media containing either 10% heat inactivated FBS or 10% charcoal-stripped heat inactivated FBS (Invitrogen). Similar results were obtained with both sources of FBS (data not shown).

*All-trans retinoic acid* (Sigma, St. Louis, MO) was reconstituted to 1 mM in DMSO. Ro41-5253 (Ro41, Enzo Life Sciences, Farmingdale, NY) was reconstituted to 10 mM in DMSO. For all experiments ATRA, Ro41, and vehicle (DMSO) were first diluted 1:1000 in 1×PBS and then added to the culture medium at the desired concentration. For HDM proliferation assays, D. Pteronyssinus extract (ALK, Round Rock TX) was used at 40 AU/mL.

### Intracellular cytokine staining (ICCS) and flow cytometry

ICCS was performed according to published methods [17]. Briefly, after activation, cells were washed once with cold PBS, labeled on ice with Live/Dead Fixable Aqua Dead Cell Stain (Invitrogen), washed, and fixed in 4% paraformaldehyde (Electron Microscopy Sciences, Hatfield, PA). Cells were then suspended in PBS with 10% DMSO (Sigma-Aldrich) and cryopreserved at -80°C. All antibodies and clones used for flow cytometry analysis have been described [17]. Samples were acquired on an LSR II flow cytometer (BD Biosciences, Franklin Lakes, NJ) and analyzed using FlowJo software (Tree Star, Ashland, OR). Typical forward versus side scatter identified lymphocytes, and cell doublets were excluded using forward scatter area versus height. After gating on viable CD3+, CD4+ cells, data were plotted for various cytokines based on absolute percentages or cell numbers within the respective gate.

### qRT-PCR

RNA was extracted from cell pellets using the RNeasy mini kit (Qiagen, Gaithersburg, MD), treated with DNase (Qiagen), and cDNA was prepared from 100–300 ng RNA using Qscript cDNA Supermix (Quanta Biosciences, Gaithersburg, MD). qRT-PCR analysis was performed using the delta Ct method of comparison on the Step-One-Plus Real-Time PCR System Thermal Cycling Block (Applied BioSystems, Carlsbad, CA). All FAM-MGB-labeled TaqMan probe and primer sets for IL-4 (Hs00166237_m1), IL-5 (Hs01548712_g1), IL-13 (Hs00174379_m1), and GAPDH (Hs_03929097_g1) were purchased from Applied Biosystems.

### Statistical analysis

Specific statistical methods used are noted in each Figure Legend. qRT-PCR fold change was calculated as; $\frac{\mathrm{Treated}\;\left(\mathrm{ATRA}\;\mathrm{or}\;\mathrm{Ro}41\right)}{\mathrm{Vehicle}\;\mathrm{control}}$_,_ when this quotient was <1, the negative of the reciprocal value was calculated as described [23].

## Results

### Reciprocal regulation of allergen-specific Th2 cytokine response by RARα modulators

ATRA enhances human Th2 cell cytokine expression in polyclonally activated T cells [6,16]. To examine the relevance of these findings to allergic disease, we analyzed the effects of ATRA and the inhibitor Ro415253 (Ro41) on house dust-mite antigen (HDM) stimulated PBMC from allergic asthmatic subjects. Cell Trace Violet (CTV) was used to track CD4+ HDM-specific memory Th2 cells, which were identified by gating on CTV^low^ cells that had proliferated in response to HDM antigen (Figure 1A). Neither RARα agonist (ATRA), nor RARα antagonist (Ro41) significantly affected the total number of CD4 T cells proliferating in response to HDM (Figure 1B). However, the output of HDM-specific proliferated IL-5+ Th2 cells was dose-dependently enhanced by ATRA, and reciprocally suppressed by Ro41 (Figure 1C, D).

We have previously characterized two major human Th2 subpopulations: IL-5- Th2 (IL-5-, IL-4+, IL-13+) and IL-5+ Th2 cells (IL-5+, IL-4+, IL-13+), which represent less and more highly differentiated Th2 cells, respectively [17]. We next analyzed HDM-expanded T cells to determine how ATRA and Ro41 affect these Th2 cell subpopulations (Figure 1E). RARα modulators reciprocally regulated Th2 cell output in the IL-5+ Th2 but not IL-5- Th2 subpopulation (Figure 1E, F).

In sum, these data demonstrate that ATRA increases the output of IL-5+ Th2 cells from allergen driven cultures.

### Intrinsic regulation of IL-5+ Th2 cell proliferation by RARα modulators

**Figure 2:**
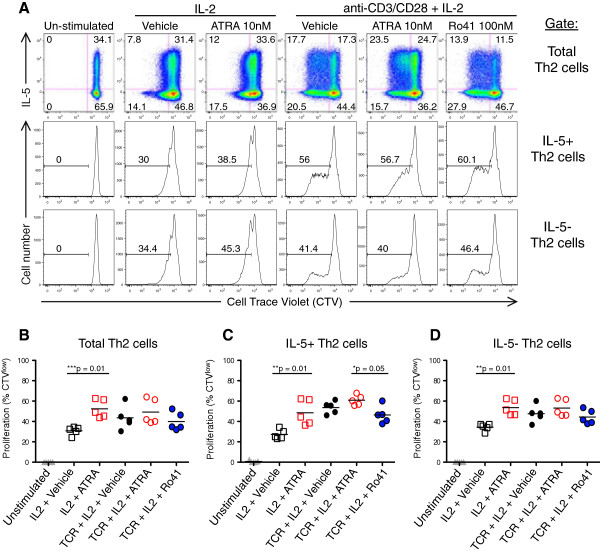
**RARα modulators intrinsically regulate IL-5+ Th2 cell proliferation.** In vitro differentiated Th2 cells were CTV labeled and activated with the noted stimuli in the presence and absence of ATRA or Ro41. After 8d cells were restimulated with PMA and ionomycin and ICCS was performed. **(A)** Representative flow plot showing total IL-5 vs. CTV (top panel), as well as CTV staining of IL-5+ Th2 (middle panel), and IL-5- Th2 (bottom panel) subpopulations. Combined results from 5 Th2 cultures showing CTV^low^ percentages of **(B)** total, **(C)** IL-5^+^ Th2, and **(D)** IL-5^-^ Th2 subpopulations. Data are presented with means (bar) and p values were generated via paired Student’s t test.

This greater output of allergen specific IL-5+ Th2 cells observed in ATRA treated samples in Figure 1 may be due to several potential mechanisms, including: enhanced Th2 differentiation, enhanced Th2 cytokine expression, preferential Th2 proliferation, or inhibition of Th2 apoptosis. To test if ATRA intrinsically regulates Th2 cell proliferation, we employed in vitro differentiated human Th2 cells and examined their proliferation after modulation with RARα agonist and antagonist treatment (Figure 2). As expected, T cell receptor (TCR) plus IL-2 stimulation induced many more generations of cell division than did IL-2 alone (Figure 2A). In the presence of IL-2 alone ATRA significantly increased Th2 cell proliferation (Figure 2B). With maximal activation (anti-CD3, CD28 plus IL-2), ATRA did not further augment Th2 cell proliferation.

We next examined whether RARα modulation differentially affected the proliferation of human Th2 subpopulations. Among in vitro differentiated Th2 cells, ATRA augmented IL-2 induced proliferation of both the IL-5+ and IL-5- subpopulations (Figure 2C, D). However, Ro41 significantly inhibited the proliferation of the IL-5+, but not the IL-5- subpopulation. This Ro41 inhibition further demonstrates the differential responsiveness of the IL-5+ vs. IL-5- Th2 subpopulations to RARα modulators.

**Figure 3:**
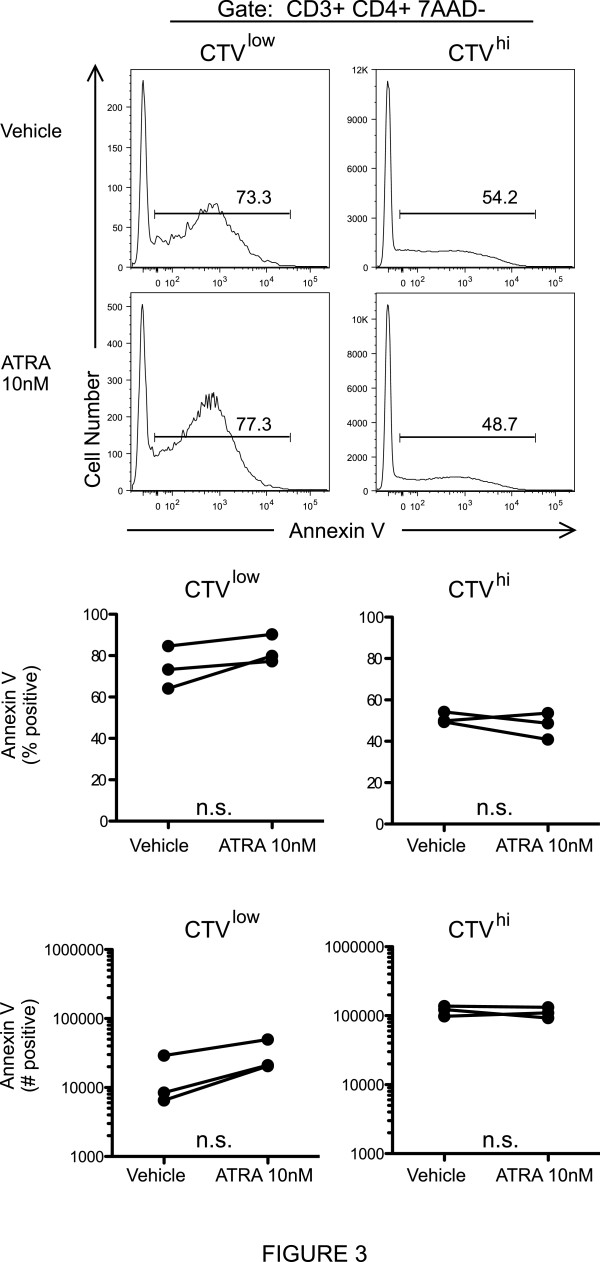
**ATRA does not affect apoptosis of HDM-expanded Th2 cells.** CTV labeled PBMCs from allergic asthmatic subjects were cultured with HDM Ag for 7d with ATRA or DMSO vehicle control. Annexin V staining was measured in the CTV^low^ (unprolferated) and CTV^high^ (proliferated HDM Ag specific) cells, after first gating on CD4+, 7AAD- lymphocytes. Above, data from a representative experiment. Below, combined results from 3 independent experiments. The percentage and number of annexin V positive cells are plotted. No statistical differences were observed by Student’s t test.

**Figure 4:**
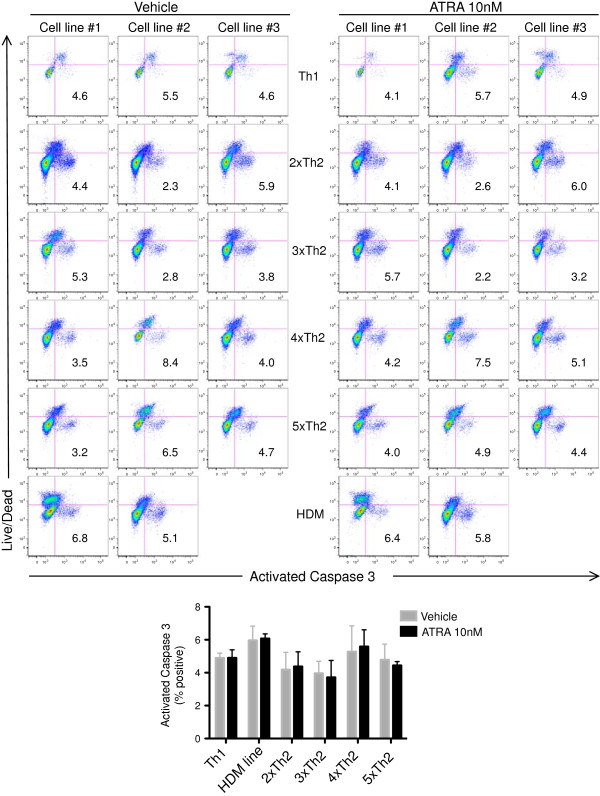
**ATRA does not affect caspase-3 activation in CD3 stimulated Th2 cells.** Th1 and Th2 cell lines were generated *in vitro* then activated with anti-CD3 antibody in presence of ATRA or DMSO vehicle control. Cultures were harvested 4d later and stained for activated caspase-3. 2xTh2, 3xTh2, etc. represent 2, and 3 serial 7d cycles of Th2 differentiation. Above, flow cytometry plots are shown for all cell lines. Below, combined results from all cultures noting the percentage of viable (Live/Dead negative) activated caspase-3+ cells (mean +/- SEM). No statistical differences were observed by paired Student’s t test.

To further address the cellular mechanisms responsible for the ATRA mediated increase in IL-5+ Th2 cell output, we examined ATRA induction of apoptosis. Notably, ATRA did not alter annexin V expression by CD4+ T cells in Th2 dominant HDM proliferation cultures (Figure 3). Additionally, ATRA did not affect caspase-3 activation in CD3 activated Th1 or Th2 cell lines (Figure 4).

In sum, these data demonstrate that ATRA positively regulates Th2 cell proliferation via T cell intrinsic mechanisms, and that RARα modulators have specificity for the IL-5+ Th2 subpopulation. Additionally, apoptosis is not playing a major role in the preferential outgrowth of IL-5+ Th2 cells induced by ATRA.

### ATRA inhibits in vitro Th2 cell differentiation

**Figure 5:**
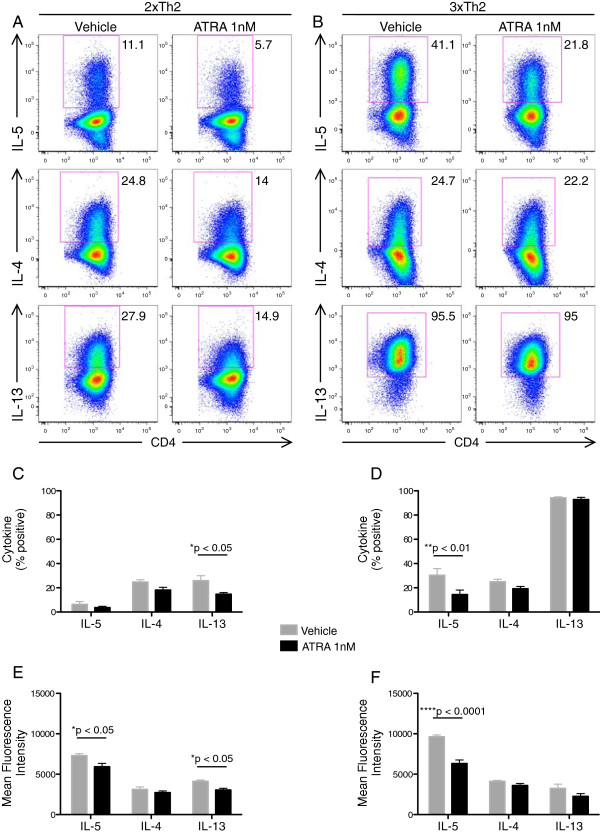
**ATRA inhibits *in vitro *Th2 cell differentiation.** Naïve CD4 T cells were isolated by MACS and differentiated under Th2 culture conditions in the presence of 1nM ATRA or DMSO vehicle control. **(A, B)** After 2 and 3 rounds of differentiation (2xTh2 and 3xTh2, respectively) cells were restimulated with PMA and ionomycin and analyzed by ICCS. **(C, D)** Percentages and **(E, F)** mean fluorescence intensity (MFI) of cytokine positive 2xTh2 (C, E) and 3xTh2 (D, F) cells are plotted from 3 experiments. Data is presented as mean +/- SEM. 2-way ANOVA was performed and Bonferroni multiple comparisons p values are displayed.

We next examined whether the pro-Th2 effects of ATRA may be due to augmentation of Th2 differentiation. We hypothesized that the addition of ATRA to in vitro Th2 cell differentiation cultures would enhance the frequency and/or kinetics of appearance of Th2 cytokines. Counter to our hypothesis, ATRA inhibited Th2 cell differentiation of both 2×Th2 and 3×Th2 cells (Figure 5).

### ATRA and Ro41 reciprocally regulate IL5, but not IL4 or IL13, gene expression

**Figure 6:**
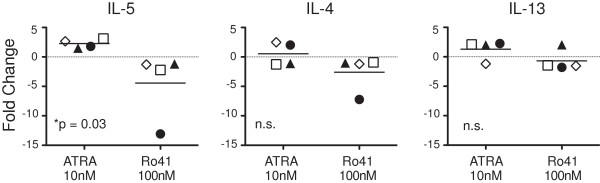
**ATRA and Ro41 reciprocally regulate IL5 gene expression.***In vitro* differentiated 2xTh2 (open symbols) and 3xTh2 (closed symbols) cells were cultured with ATRA, Ro41, or DMSO vehicle for 6hrs. The expression of IL-5, IL-4, and IL-13 were determined by qRT-PCR and fold change (treated/vehicle) plotted. Th2 cell lines stimulated in the presence of IL-2 (50 U/ml) only. Data points represent individual Th2 cell lines. Paired Student’s t test was used to evaluate the data. Each symbol represents a specific cell line.

We next tested the hypothesis that RARα modulators directly regulate Th2 cytokine gene expression via Th2 cell intrinsic mechanisms. IL-2 activation (in the absence of TCR signals) increased expression of IL5 message, which was significantly modulated by ATRA and Ro41, in a reciprocal manner (Figure 6). No differences were observed in IL4 or IL13 gene expression. These data demonstrate that in addition to their effects on IL-5+ Th2 cell proliferation, RARα modulators directly regulate IL5 gene expression.

## Discussion

Here we report that the pro-Th2 effect induced by retinoic acid is primarily a direct result of Th2 cell-intrinsic regulation of the cytokine IL-5 by RARα. Additionally, these data demonstrate that RARα regulates the proliferation of IL-5+ but not IL-5- Th2 cells. These results imply that the highly differentiated IL-5+ effector Th2 cell subpopulation is the primary Th2 cell population affected by RARα modulators; in contrast, the less differentiated IL-5- Th2 cells were significantly less affected. Both the proliferation of IL-5+ Th2 cells as well as IL5 gene expression was suppressed by the RARα antagonist Ro41, which suggests that RARα antagonism might provide a therapeutic approach to inhibit the function of pathogenic pro-inflammatory IL-5+ Th2 cells.

The ATRA/RARα pathway is a well-known inducer of Th2 cytokine responses both in vitro and in vivo, working through both T cell intrinsic and extrinsic mechanisms [6,16,24]. Our results provide evidence, that among the three major Th2 cytokines, RARα modulators predominantly regulate IL-5 expression. We and other groups have recently characterized IL-5+ Th2 cells as a more highly-differentiated Th2 subpopulation with greater pro-inflammatory function [17-19]. This current work demonstrates that some of the pro-Th2 activity of ATRA is due to increases in Th2 proliferation, particularly that of the IL-5+ Th2 subpopulation. Notably, despite this overall pro-IL-5 activity, ATRA did not enhance Th2 differentiation (Figure 5).

Notably, whereas ATRA promoted IL-5+ Th2 responses, the RARα antagonist Ro41 actually inhibited IL-5+ Th2 responses. Such inhibition may be due to Ro41 acting as a neutral antagonist blocking RARα activation by endogenous ATRA in the cell culture media or by Ro41 acting as an inverse agonist [25]. Notably, the latter activity has not been previously reported for Ro41.

Previous human studies showing ATRA-induced Th2 cytokine production have utilized PBMC or CD4 T cells activated with polyclonal stimuli [6,16]. This study is notable for using allergen specific Th2 cells from allergic asthmatic subjects as well as highly differentiated Th2 cell lines. The use of such pathogenically relevant Th2 cells lines confirm and extend previous observations using mitogen activated PBMC from healthy donors.

This current work showing ATRA augmentation of pathogenic allergen specific Th2 responses underscores the potential clinical relevance of these findings. RA agonists are available both as prescription and over the counter formulations; these data suggest that RA supplementation may potentially augment Th2 responses and thus promote allergic disease, as observed in the mouse model of asthma [15]. Alternatively, other pathways may augment local ATRA levels by the upregulation of retinaldehyde dehydrogenase 2, which is required for the biosynthesis of retinoic acid. To that end, Shreffler and colleagues have characterized a previously undescribed peanut protein that upregulates retinaldehyde dehydrogenase 2 in myeloid dendritic cells [26].

We studied the intrinsic regulation of Th2 cell function (proliferation and cytokine production) by reactivating highly differentiated Th2 cells. Since this culture system utilizes APC-free in vitro differentiated Th2 cells, the ATRA/Ro41-mediated effects are, by definition, mediated by Th2 cell intrinsic mechanisms. Using this APC-free system, ATRA did not augment Th2 differentiation, suggesting that the pro-Th2 effects of RARα modulation are not through enhanced T cell-intrinsic effects on Th2 differentiation.

Using either Annexin V or activated caspase 3 to identify apoptotic cells, RARα modulators did not affect the frequency of apoptosis (Figures 3 and 4). Taken together, these findings suggest that ATRA augments Th2 responses by promoting Th2 cell proliferation and gene expression and not through differential modulation of cell death or apoptosis.

An inherent limitation of studies using pharmacological inhibitors is the potential for off-target effects. Indeed, Ro41 has been shown to activate peroxisome proliferator activated receptor-γ (PPAR-γ) at concentrations of 1 μM [27], which is 10-fold greater than the concentrations used in this study. To further address whether off-target effects of Ro41 on PPAR-γ could have been responsible for its inhibition of IL-5+ Th2 proliferation, we examined the effect of the PPAR-γ agonist GW7845 on Th2 cultures. GW7845 did not have any effect on IL-4, IL-5 or IL-13 expression in these cultures (data not shown). The relatively low concentration of Ro41 used in this study as well as the lack of effect of PPAR-γ activators, make it unlikely that Ro41 was acting through off-target effects.

**Figure 7:**
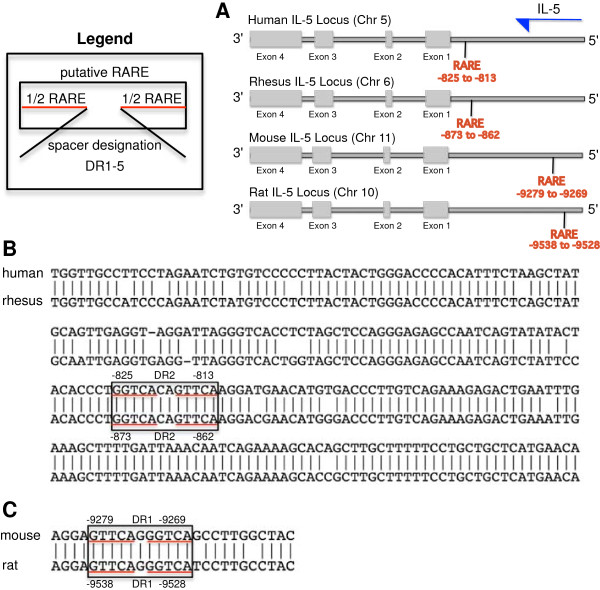
**The human IL-5 promoter (0 to -1054) contains a putative retinoic acid response element (RARE).** A RARE contains two RARE ½ sites [G(T/G)TCA] and from 1 to 5 spacer nucleotides designated by the “DR” designation. **(A)** A Schematic representation of the IL-5 locus between species. The putative RARE location in the IL5p is compared between human, mouse, rat, and rhesus. Alignment of DNA sequence around the IL5p putative RARE between **(B)** human and rhesus, and **(C)** mouse and rat.

This apparent direct regulation of Th2 cytokine gene expression by RARα prompted us to examine if a putative retinoic acid response element (RARE) exists in the promoter regions of Th2 cytokine genes (*IL5p*, *IL4p*, *IL13p*). We thus analyzed the 10 kb genomic DNA sequence of the human IL5, IL4, and IL13 promoters using the University of California Santa Cruz genome browser. We identified a single putative RARE (5′-TGGTCACAGTTCA-3′) in the human *IL5p* (-825 to -813) but not in the human *IL4p* and *IL13p*, suggesting that IL5 could be a RARE-responsive gene. The genomic location of the *IL5p* putative RARE is comparable between human and rhesus, and similarly, between mouse and rat (Figure 7A). The *IL5p* putative RARE sequence is identical between human and rhesus (Figure 7B) and similarly, between mouse and rat (Figure 7C). Subsequent studies are needed to verify if this putative RARE is functionally active.

## Conclusions

In conclusion, we demonstrate that RARα modulators act on Th2 cells through multiple mechanisms, including Th2 cell intrinsic augmentation of proliferation and IL-5 expression. In all experiments, the magnitude of the effect was most apparent for IL-5 responses. The potent induction by ATRA and reciprocal inhibition of IL-5 by Ro41 supports that these effects are mediated through the RARα receptor. These data demonstrate that RARα modulation has a major impact on human Th2 responses and suggests that RARα may be a potential therapeutic target for anti-Th2 therapy.

## Abbreviations

RA: Retinoic acid; ATRA: All-trans retinoic acid; RARα: Retinoic acid receptor alpha; RXR: Retinoid X receptor; PBMC: Peripheral blood mononuclear cells; HDM: House dust mite; Ag: Antigen; APC: Antigen presenting cells; TCR: T cell receptor; CTV: Cell trace violet; Th2: T helper 2; Ro41: Ro41-5253; RARE: Retinoic acid response element; IL5p: Interleukin 5 gene promoter; IL13p: Interleukin 13 gene promoter; IL4p: Interleukin 4 gene promoter; mRNA: Messenger ribonucleic acid; ICCS: Intracellular cytokine staining; qRT-PCR: Quantitative real time PCR; DMSO: Dimethyl sulfoxide; DMEM: Dulbecco modified eagles medium; FBS: Fetal bovine serum; ANOVA: Analysis of variation; MFI: Mean fluorescence intensity; SEM: Standard error of mean

## Competing interests

The authors declare that they have no competing interests.

## Authors’ contributions

CP conceived of the study. YY performed initial pilot experiments. DLW carried out the experiments. All authors contributed to the design and analysis of the study. DLW and CP prepared the manuscript and performed statistical analysis. All authors read and approved the final manuscript.
